# Acute intramyocardial lipid accumulation in rats does not slow cardiac conduction per se

**DOI:** 10.14814/phy2.14049

**Published:** 2019-04-09

**Authors:** Christa F. Jensen, Emil D. Bartels, Thomas H. Braunstein, Lars B. Nielsen, Niels‐Henrik Holstein‐Rathlou, Lene N. Axelsen, Morten Schak Nielsen

**Affiliations:** ^1^ Department of Biomedical Sciences Faculty of Health Sciences University of Copenhagen Copenhagen Denmark; ^2^ Department of Clinical Biochemistry Copenhagen University Hospital Rigshospitalet Copenhagen Denmark

**Keywords:** Cardiac clectrophysiology, conduction velocity, fasting, lipid accumulation, palmitate, triglycerides

## Abstract

Diabetic patients suffer from both cardiac lipid accumulation and an increased risk of arrhythmias and sudden cardiac death. This correlation suggests a link between diabetes induced cardiac steatosis and electrical abnormalities, however, the underlying mechanism remains unknown. We previously showed that cardiac conduction velocity slows in Zucker diabetic fatty rats and in fructose‐fat fed rats, models that both exhibit prominent cardiac steatosis. The aim of this study was to investigate whether acute cardiac lipid accumulation reduces conduction velocity per se. Cardiac lipid accumulation was induced acutely by perfusing isolated rat hearts with palmitate‐glucose buffer, or subacutely by fasting rats overnight. Subsequently, longitudinal cardiac conduction velocity was measured in right ventricular tissue strips, and intramyocardial triglyceride and lipid droplet content was determined by thin layer chromatography and BODIPY staining, respectively. Perfusion with palmitate‐glucose buffer significantly increased intramyocardial triglyceride levels compared to perfusion with glucose (2.16 ± 0.17 (*n* = 10) vs. 0.92 ± 0.33 nmol/mg WW (*n* = 9), *P* < 0.01), but the number of lipid droplets was very low in both groups. Fasting of rats, however, resulted in both significantly elevated intramyocardial triglyceride levels compared to fed rats (3.27 ± 0.43 (*n* = 10) vs. 1.45 ± 0.24 nmol/mg WW (*n* = 10)), as well as a larger volume of lipid droplets (0.60 ± 0.13 (*n* = 10) vs. 0.21 ± 0.06% (*n* = 10), *P* < 0.05). There was no significant difference in longitudinal conduction velocity between palmitate‐glucose perfused and control hearts (0.77 ± 0.025 (*n* = 10) vs. 0.75 m/sec ± 0.029 (*n* = 9)), or between fed and fasted rats (0.75 ± 0.042 m/sec (*n* = 10) vs. 0.79 ± 0.047 (*n* = 10)). In conclusion, intramyocardial lipid accumulation does not slow cardiac longitudinal conduction velocity per se. This is true for both increased intramyocardial triglyceride content, induced by palmitate‐glucose perfusion, and increased intramyocardial triglyceride and lipid droplet content, generated by fasting.

## Introduction

Diabetes is a worldwide problem and an important cause of cardiovascular disease. Some cardiac complications may arise secondary to coronary atherosclerosis, but even in the absence of vascular alterations, the aberrant lipid metabolism of the diabetic heart affects cardiac structure and function (reviewed in (Carpentier 2018)). Supporting an independent role of diabetes in cardiac disease, diabetic patients are at increased risk of sudden cardiac death even when corrected for known cardiac disease (reviewed in (Zaccardi et al. 2014)). Although the underlying causes are not entirely clear, diabetic patients exhibit electrophysiological disturbances such as QT prolongation (Brown et al. 2001; Veglio et al. 2002; Isaksen et al. 2018a) and dispersion (Veglio et al. 2002), altered T wave morphology (Isaksen et al. 2018b) and QRS prolongation (Yang et al. 1990; Isaksen et al. 2018a).

QRS prolongation may arise from disturbed conduction pathways or reduced conduction velocity. Several models of diabetes exhibit conduction slowing, but with differences in the underlying mechanisms. For models of type 1 diabetes mellitus, changes in connexin 43 expression (Lin et al. 2006; Stilli et al. 2007; Howarth et al. 2008), phosphorylation (Lin et al. 2006, 2008; Howarth et al. 2008), and localization (Lin et al. 2006; Nygren et al. 2007; Shimoni et al. 2009), as well as development of fibrosis (Regan et al. 1977; Bakth et al. 1986) were suggested to explain slowed conduction and/or increased susceptibility to arrhythmias. Also Na^+^‐currents (Stables et al. 2014) and K^+^‐currents, (reviewed in (Gallego et al. 2009)) are altered in diabetic animals, and simulation studies indicate that synergistic effects of several pathological mechanisms may explain conduction disturbances in diabetic models (Ghaly et al. 2010).

Less is known about the mechanisms underlying conduction slowing in type 2 diabetes. We have investigated the type 2 Zucker diabetic fatty (ZDF) rat (Olsen et al. 2013), and the prediabetic fructose‐fat fed rat (Axelsen et al. 2010a) which both show a decreased cardiac conduction velocity ex vivo. Hearts from fructose‐fat fed rats also have QRS prolongation in vivo, as well as a higher risk of ventricular fibrillation following ischemia‐reperfusion in isolated hearts (Axelsen et al. 2015). In ZDF rats, the conduction slowing was not explained by either changes in connexin 43 expression, cell size, or fibrosis (Olsen et al. 2013), factors that are closely associated with conduction slowing. As for the ZDF rats, we also investigated connexin 43 expression and localization, as well as levels of fibrosis and cell size in the fructose‐fat fed rats. These studies were extended with in vitro measurements of gap junctional coupling as well as Na^+^‐ and K^+^‐currents, however, none of the parameters were changed in the fructose‐fat fed rats (Axelsen et al. 2015), and could therefore not explain the observed conduction slowing.

Beside similar conduction slowing, both ZDF rats (Zhou et al. 2000; Szczepaniak et al. 2003; Sharma et al. 2004; Olsen et al. 2013) and fructose‐fat fed rats (Axelsen et al. 2010b) exhibit cardiac steatosis. Steatosis‐related lipotoxicity plays an important role in diabetic cardiomyopathy (reviewed by (van de Weijer et al. 2011)), and correlates with both diastolic (Nielsen et al. 2002; van der Meer et al. 2007, 2008; Hammer et al. 2008a,b), and systolic dysfunction (Zhou et al. 2000; Chiu et al. 2001; Nielsen et al. 2002; Sharma et al. 2004; Suzuki et al. 2009), as well as impaired longitudinal strain and strain rate (Ng et al. 2010). In addition, intramyocardial lipid accumulation has been proposed to cause electrophysiological complications.

Lin and colleagues, showed that incubating left atrial myocytes with adipocytes, directly affected both action potential duration and resting membrane potential, as well as induced higher incidence of triggered beats (Lin et al. 2012). Along the same lines, Kang and colleagues found that long‐chain polyunsaturated fatty acids reduced membrane excitability in neonatal rat cardiomyocytes (Kang et al. 1995). Haim and colleagues found a reduced action potential duration in isolated ventricular cardiomyocytes exposed to palmitate (Haim et al. 2010). These rapid electrophysiological effects of fatty acids were reported in isolated cells, but acute effects have also been demonstrated in vivo by Hexeberg and colleagues who observed arrhythmias in rabbits infused with lipids (Hexeberg et al. 1995). Lim et al. (2011) also showed increased vulnerability to stress‐induced fibrillation and cardiac arrest in mutant *Drosophila* with increased cardiac triglyceride levels. It is difficult to specify exactly what constitutes the pathological mechanisms behind lipid induced electrophysiological disturbances based on these studies. It could be lipotoxic metabolites developing secondary to fatty acid metabolism, or alternatively, that the mere presence of lipids could increase intracellular electrical resistance directly and impede propagation. In either case, the question whether lipids acutely affect cardiac conduction has never been addressed.

The aim of this study was therefore to gain knowledge about the possible direct relationship between cardiac conduction disturbances and intramyocardial lipid accumulation. Longitudinal cardiac conduction velocity, triglyceride, and lipid droplet content were investigated in rats, either after palmitate‐glucose perfusion of isolated hearts (acute model of lipid accumulation), or after an overnight fast (subacute model of lipid accumulation).

## Methods

### Animals

The Animal Experiments Inspectorate of the Danish Ministry of Justice approved all experiments, and the study was performed in accordance to the European Parliament Directive on the Protection of Animals Used for Scientific Purposes (2010/63/EU). Male Sprague–Dawley rats (Taconic, Ll. Skensved, Denmark) were used for all experiments.

### Study design

#### Acute lipid loading by palmitate‐glucose perfusion

Hearts from rats receiving normal chow (5% fat, no. 1319 FORTI; Altromin, Lage, Germany) were isolated and perfused with either palmitate‐glucose buffer (*n* = 10) or glucose buffer (*n* = 9) for 110 min in a Langendorff setup as described in (Axelsen et al. 2015). Subsequently longitudinal conduction velocity was measured as previously described in (Axelsen et al. 2015) and intramyocardial lipid content was determined.

#### Subacute lipid loading by overnight fasting

Rats receiving normal chow, were either fasted overnight for 16–19 h (*n* = 10) or fed unlimited normal chow (*n* = 10). Tail vein fasting blood glucose levels were measured prior to cardiac tissue preparation for longitudinal conduction velocity and intramyocardial lipid determinations.

### Experimental Procedures

#### Anesthesia

Prior to heart isolation, rats were anesthetized in a 5% isoflurane (Baxter, Allerød, Denmark) filled container, and transferred to an isoflurane inhalation mask. Isoflurane was delivered in 35% O_2_ and 65% N_2_, flow = 3 L/min, and the inhalation mask was connected to a respirator (rate = 70 strokes/min, tidal volume = 3–4 mL).

#### Langendorff perfusion

When fully anesthetized, a tracheostomy was performed to maintain ventilation and anesthesia during thoracotomy. The aorta was exposed and cannulated. Hearts were quickly dissected, transferred to the Langendorff setup (Hugo Sachs) and perfused at ~80 mmHg with a 37°C warm Krebs–Henseleit (KH) buffer (in mmol/L: 118 NaCl, 4.7 KCl, 1.2 KH_2_PO_4_, 1.2 MgSO_4_7H_2_O, 1.75 CaCl_2_2H_2_0, 25 NaHCO_3_ and 11 glucose), equilibrated with carbogen (95% O_2_ ‐ 5% CO_2_). To assess contractile function, a latex balloon connected to a pressure transducer (Hugo Sachs Electronic) was placed in the left ventricle via the left atrium. A 37°C buffer filled bath was placed around the hearts. The hearts were perfused for 10 min in KH buffer followed by 110 min perfusion with either a palmitate‐glucose KH buffer: KH buffer + 1.2 mmol/L palmitic acid, 1.5 mmol/L Na_2_CO_3_, 3% bovine serum albumin (BSA) (Equitech‐Bio, Inc. Kerrville, Texas), or a glucose KH buffer: KH buffer + 3% BSA. BSA:palmitate buffer was prepared as described in (Lopaschuk and Barr 1997). Coronary flow and left ventricular developed pressure (LVDP) were recorded using IOX 2 (EMKA technologies), from which heart rate (HR), maximum increase in pressure over time during contraction (d*P*/d*t*_max_), and maximum decrease in pressure over time during relaxation (−d*P*/d*t*_max_) were derived.

#### Tissue preparations for conduction velocity, intramyocardial lipid, and water content

In fasted and fed rats a thoracotomy was performed and the hearts were quickly removed and immersed in oxygenated HEPES‐Tyrodes buffer (HT buffer), with a pH of 7.4. (in mmol/L: 136 NaCl, 4 KCl, 5 HEPES, 5 MES, 0.8 MgCl_2_, 1.8 CaCl_2_, and 10 glucose). Langendorff perfused hearts were likewise removed from the setup and immersed in oxygenated HT buffer.

Strips of right ventricular tissue were dissected in a Petri‐dish with HT buffer and a loop was sutured to each end (6‐0 mersilk, Ethicon, Johnson‐Johnson Intl, Bruxelles, Belgium). In a fluid chamber (1 mL), with the endocardium facing upward, the tissue was connected to a force‐transducer (Isometric force‐transducer, Kent, Torrington, USA) and to a stationary hook at the basal‐ and apex‐end, respectively (Olsen et al. 2011). The chamber was continuously perfused with oxygenated HT buffer (temp = 34–36°C, flow = 2 mL min^−1^) using a roller‐pump (Ole Dich, Instrumentmakers ApS, Denmark).

For some hearts two additional right ventricular tissue strips were dissected. One strip was immediately fixed using a Zamboni protocol as previously described (Axelsen et al. 2010a), and used for lipid droplet determination. The other strip was frozen in liquid nitrogen and stored at −80°C for subsequent thin layer chromatography (TLC) analysis of triglycerides. Finally, a strip from the left ventricle was dissected from some hearts for dry‐weight wet‐weight ratio determination.

#### Conduction velocity recordings

The strip located in the fluid chamber was paced with a unipolar plastic coated steel wire (diameter 0.1 mmol/L), at 2 Hz (impulse duration of 0.5 msec). The voltage (Master‐8 stimulator, A.M.P.I., Jerusalem, Israel) was set to double threshold value throughout the experiments. Strips were stretched to determine maximal force (*F*_max_) and the length was adjusted to ½ *F*_max_. The tissue was subsequently allowed to rest for 20–30 min.

By use of an isometric force‐transducer, developed force, and diastolic force was determined.

To measure longitudinal conduction velocity, two Platinum/Iridium electrodes (PI20030.5B10, Micro Probe Inc., Gaithersburg, USA), were placed on the longitudinal axis of a muscle fiber bundle. The signals were amplified by Iso‐DAM8A amplifier (World Precision Instruments, Sarasota, USA), band‐pass filtered at 300–10,000 Hz and sampled at 30 kHz (Digidata 1322A, Axon Instruments, Union City, USA). Conduction velocity was measured over a 10‐min period.

After finishing the conduction velocity protocol, the tissue strip weight and length were measured. Muscle volume (*V*) was determined by mass (*m*), divided by a density (*δ*) of 1063 mg/mm^3^: *V* = m/*δ*. Cross‐sectional area (*A*) was calculated by dividing muscle volume by muscle length (*L*): *A* = *V*/*L*. Cross‐sectional area was used to normalize developed force and diastolic force.

Time of local activation under the first and second microelectrode was determined as the time of minimum d*V*/d*t* by a custom written MatLab script. Conduction velocity was calculated as the interelectrode distance divided by the interelectrode delay.

#### Myocardial triglyceride extraction and quantification by TLC

As described in (Bartels et al. 2002) heart biopsies were weighed and subsequently minced with scissors in 0.5 mL methanol. 2 mL chloroform and 0.5 mL methanol were added and the mixture was incubated at 4°C overnight. After thorough mixing, 1 mL methanol was added and the mixture was vortexed and centrifuged for 15 min at 4°C at 2000*g*. The supernatant (lipid extract) was transferred to a new vial. The remaining tissue extract was washed twice in 0.5 mL chloroform and 0.5 mL methanol followed by centrifugation and transfer of the supernatant to the lipid extract vial. Lipid extracts were washed ad modum Folch (Folch et al. 1957). After evaporation of solvents under N_2_, the lipids were dissolved in toluol (3 *μ*L/mg wet weight of tissue). 1 *μ*L of each sample were added on a TLC plate (DC‐fertigplatten SIL G‐25, Macherey‐Nagel, Duren, Germany; 20 × 20 cm) that was precleaned in acetone and preactivated by a 110°C heating for 30 min. Lipid standards (Sigma, Vallensbaek Strand, Denmark) with defined lipid amounts were also applied. The plates were immersed first in Hexan:Diethylester:Acetic acid (70:30:2) and then in Hexan, before being stained with 10% cupric sulfate (wt/vol) in 8% phosphoric acid (vol/vol), dried, and baked for 2 min at 200°C. Plates were scanned in a HP Scanjet 4890 and lipid content was quantified by digital image analysis using the ImageJ software. On a separate TLC plate, all samples were analyzed in duplicate.

#### BODIPY staining and intramyocardial lipid droplet content

Quantification of intramyocardial lipid droplets was done by fixing small fragments of right ventricular tissue in Zamboni (as described in (Axelsen et al. 2010a)), after which the tissues were stained with BODIPY‐493/503 (Invitrogen) and analyzed by confocal microscopy (as described in (Prats et al. 2006)).

Bundles of myocardial tissue were isolated under a stereomicroscope using fine forceps and incubated with BODIPY‐493/503 (20 *μ*L in 1 mL PBS) for 30 min at room temperature. Subsequently, the samples were washed in PBS three times for 10 min each. Myocardial bundles were mounted in Vectashield media, (Vector Laboratories, Burlingame, CA) on a glass slide and analyzed by confocal microscopy (Zeiss LSM 780 or Zeiss LSM 700). For each sample, stacks of 14 images (step size 0.39 *μ*m) (covering 5.03 *μ*m in the z‐plane) with the x‐y dimensions of 56 × 56 *μ*m were recorded at six different randomly chosen regions using a Zeiss 63x NA 1.4 oil immersion objective.

Images were analyzed using the ImageJ software. Images were smoothed and processed with a Laplacian differential filter, after which they were smoothed four more times to level out differences in gray levels over the lipid droplets. Hereafter, the stacks were thresholded in order to remove the tissue autoflorescence. The resulting stacks were then processed with the 3D Objects Counter (FiJi plugin) to obtain droplet density and volumes. During volume calculations, all objects found only in one image was considered to be background and therefore omitted from analysis.

To determine cardiomyocyte volume, images were smoothed twice and thresholded to include tissue autofluorescense. Using a macro to calculate the percentage tissue coverage in each image in the stacks, tissue volume was calculated by multiplying the average percentage of tissue for a whole stack with the entire recorded volume.

All postprocessing of the images was done in a blinded fashion.

#### Chemicals

Chemicals were purchased at Sigma‐Aldrich Danmark A/S (Brøndby, Denmark).

#### Statistics

Data are reported as mean ± standard error of the mean (SEM). Differences between groups were analyzed using Student's *t*‐test (two‐sided, unpaired, equal variances). Quantification of lipid droplets from Langendorff‐perfused hearts was performed in duplicate. Cardiac water content was compared using one‐way analysis of variance (ANOVA) followed by a Bonferroni's multiple comparisons test. Langendorff data were analyzed using two‐way repeated measures ANOVA. Where appropriate, a Bonferroni post hoc test was used to determine statistical differences between groups at individual time points. GraphPad Prism (GraphPad Software, La Jolla, CA) was used for statistical calculations. *P* < 0.05 was considered statistically significant.

## Results

### Acute lipid loading

#### Animal characteristics

There was no significant difference in the weight of the rats used for either palmitate‐glucose (352.20 ± 9.34 g, (*n* = 10)) or glucose (354.33 ± 10.61 g, (*n* = 9)) perfusion.

#### Mechanical function of Langendorff‐perfused heart

**Figure 1:**
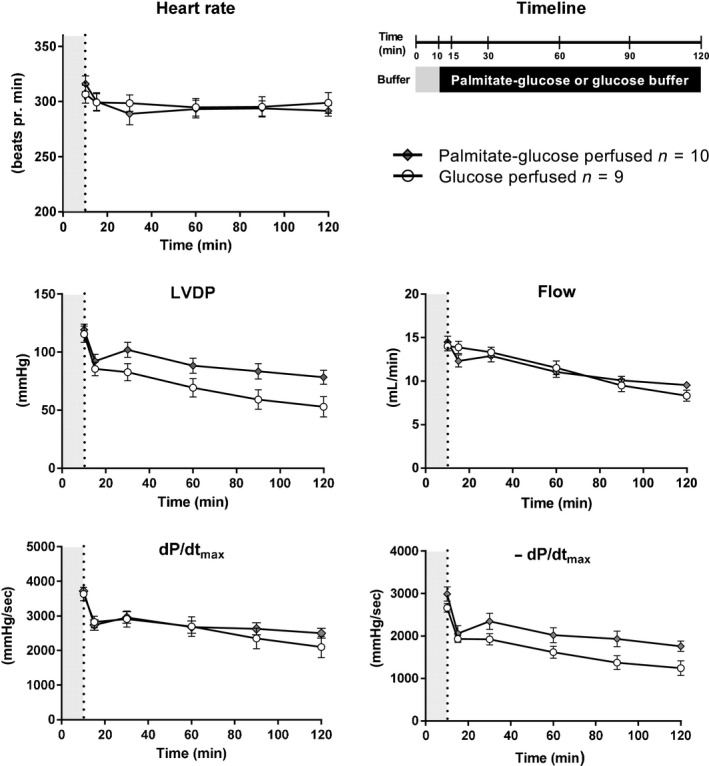
Langendorff data. Hearts were initially perfused for 10 min with normal Krebs–Henseleit buffer (gray on timeline and on graphs) after which the buffer was switched to a palmitate‐glucose or glucose buffer for 110 min (black on timeline). The graphs depict heart rate, left ventricular developed pressure (LVDP), flow, maximal change in pressure over time during systole (d*P*/d*t*_max_) and maximal change in pressure over time during diastole (−d*P*/d*t*_max_) as a function of time for both palmitate‐glucose and glucose‐perfused hearts. The graphs were conducted by taking the average values for 1 min at six different time points (10, 15, 30, 60, 90, and 120 min). Statistical analysis was only performed for time points 15–120 min, as this is where the group specific buffers were in use. Values are presented as means ± SEM. For LVDP, flow, d*P*/d*t*_max_, and −d*P*/d*t*_max_ there was a statistically significant decrease over time, as well as a significant interaction between time and buffer type.

Figure 1 shows the development of HR, LVDP, d*P*/d*t*_max_, −d*P*/d*t*_max_, and flow over time. After 10 min, the perfusion was switched from a standard KH buffer to either palmitate‐glucose or glucose buffer. Irrespective of buffer type, most parameters (except flow) decreased rapidly within the first 5 min after buffer switch, most likely due to buffering of free calcium by the BSA content of the buffers. During the rest of the perfusion period, HR remained stable, whereas all mechanical parameters as well as flow tended to decline. A repeated measures ANOVA showed a significant interaction between time and perfusate for LVDP, d*P*/d*t*_max_, −d*P*/d*t*_max_ and flow. The significant interaction indicates that the time evolution of the four parameters differed between the palmitate‐glucose and glucose‐perfused hearts. However, no individual time points were significantly different between palmitate‐glucose and glucose‐perfused hearts for any parameter, indicating that the perfusate had only a minor influence on cardiac function.

In this study, Langendorff perfusion itself induced edema with significantly higher water content measured in perfused hearts compared to freshly excised hearts (79.5 ± 0.2 (*n* = 16) vs. 76.8 ± 0.7% (*n* = 15) *P* <0.0001). There was, however, no difference in cardiac water content between the palmitate‐glucose and glucose‐perfused hearts (79.6 ± 0.2 (*n* = 8) vs. 79.4 ± 0.3% (*n* = 8), NS).

#### Intramyocardial lipid levels

**Figure 2:**
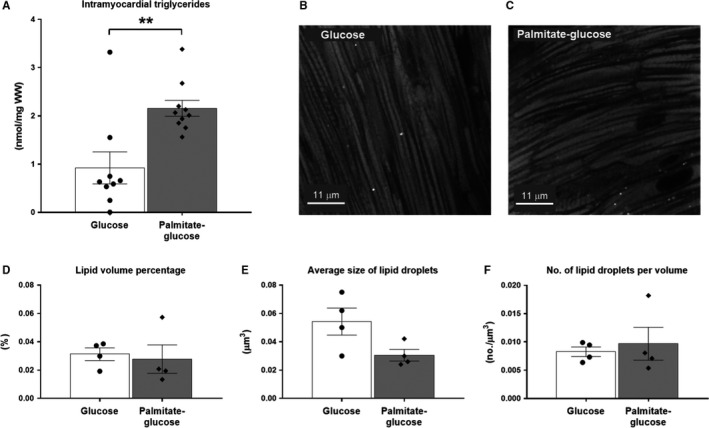
Intramyocardial lipid content in Langendorff perfused hearts. (A) Intramyocardial triglyceride content in glucose (*n* = 9) and palmitate‐glucose (*n* = 10) perfused hearts. (B and C) Representative images of intramyocardial lipid droplets in glucose and palmitate‐glucose perfused hearts, respectively (white dots represent lipid droplets stained with Bodipy‐493/503). (D) Quantitative assessment of intramyocardial lipid volume percentage, (E) average size of intramyocardial lipid droplets, (F) average number of lipid droplets per volume, in glucose (*n* = 2) and palmitate‐glucose (*n* = 2) perfused hearts. Cardiac tissue from the two animals in each group were stained for lipid droplets and quantified in duplicates (explaining the 4 points on each column in figure D, E, and F). Values are presented as means ± SEM. **P < 0.01.

Palmitate‐glucose perfusion significantly increased intramyocardial triglyceride levels compared to hearts perfused with glucose alone (2.16 ± 0.17 nmol/mg WW (*n* = 10) vs. 0.92 ± 0.33 nmol/mg WW (*n* = 9), *P* < 0.01) (Fig. 2A). Both groups showed very low levels of lipid droplets (Fig. 2B and C), and there were no significant differences in either lipid volume (Fig. 2D), or average size and number of lipid droplets (Fig. 2E–F) between palmitate‐glucose and glucose‐perfused hearts. In order to confirm these findings, lipid droplet staining and quantification were performed in duplicates. The low levels of lipid droplets indicate that the elevated triglyceride levels in palmitate‐glucose perfused hearts did not result in the formation of lipid droplets within the timeframe of the experiments.

#### Electrophysiological and mechanical function in right ventricular tissue strips

**Figure 3:**
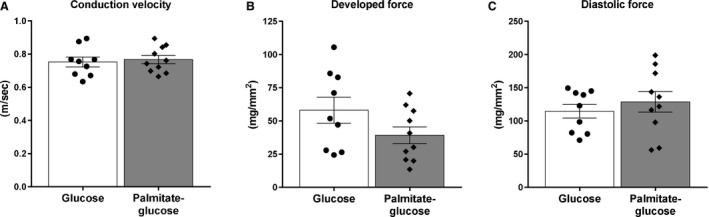
Ventricular longitudinal conduction velocity, developed force, and diastolic force in Langendorff perfused hearts. (A) Conduction velocity, (B) developed force, and (C) diastolic force was measured in the longitudinal direction of right ventricular strips from rat hearts initially perfused with either glucose (*n* = 9) or palmitate‐glucose (*n* = 10) buffer in a Langendorff setup for 110 min. Values are presented as means ± SEM.

There was no significant difference in longitudinal conduction velocity between palmitate‐glucose and glucose‐perfused hearts (0.77 ± 0.025 m/sec (*n* = 10) vs. 0.75 m/sec ± 0.029 (*n* = 9), NS) (Fig. 3A). Mechanical function was also not different between palmitate‐glucose and glucose‐perfused hearts, when comparing either developed pressure (39.27 ± 6.29 mg/mm^2^ (*n* = 10)) vs. (58.08 ± 9.87 mg/mm^2^ (*n* = 9), NS) (Fig. 3B), or diastolic force (128.8 ± 15.47 mg/mm^2^, (*n* = 10) vs. (114.6 ±10.49 mg/mm^2^ (*n* = 9), NS) (Fig. 3C).

### Subacute lipid loading

#### General characteristics of fasted rats

Fasting rats overnight significantly reduced their body weight (299.4 ± 6.45 g (*n* = 10) vs. 324.1 ± 7.32 g (*n* =10), *P* < 0.05) and blood glucose level (4.24 ±0.15 mmol/L (*n* = 10) vs. 5.40 ± 0.36 mmol/L, (*n* = 10), *P* < 0.01) compared to rats fed ad libitum.

#### Intramyocardial lipid levels

**Figure 4:**
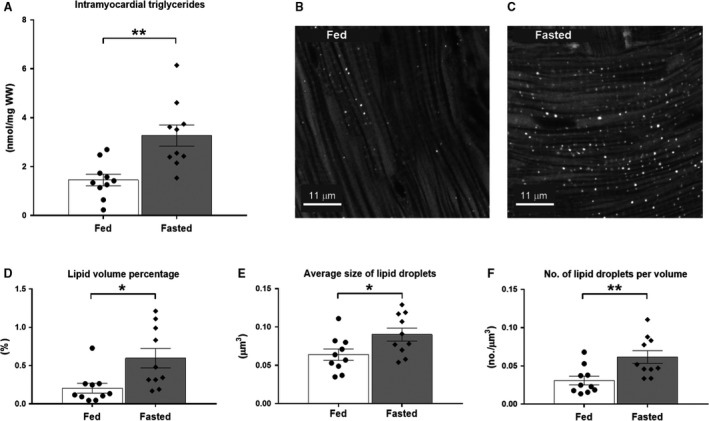
Intramyocardial lipid content in hearts from fed and fasted rats. (A) Intramyocardial triglyceride content in hearts from rats either fed normal chow ad libitum (*n* = 10) or fasted overnight (*n* = 10). (B and C) Representative images of intramyocardial lipid droplets in fed and fasted hearts, respectively (white dots represent lipid droplets stained with Bodipy‐493/503). (D) Quantitative assessment of intramyocardial lipid volume percentage, (E) average size of intramyocardial lipid droplets and (F) average number of lipid droplets per volume, in hearts from fed (*n* = 10) or fasted (*n* = 10) rats. Values are presented as means ± SEM. *P < 0.05, **P < 0.01.

Fasting rats overnight significantly increased the intramyocardial lipid content (Fig. 4). Intramyocardial triglyceride levels were significantly increased compared to rats fed normal chow ad libitum (3.27 ± 0.43 nmol/mg WW (*n* = 10) vs. 1.45 ± 0.24 nmol/mg WW (*n* = 10), *P* < 0.01) (Fig. 4A). As depicted in Figure 4B and C, fasting significantly increased the volume of intramyocardial lipid droplets compared to hearts from fed rats (0.60 ± 0.13% (*n* = 10) vs. 0.21 ± 0.06% (*n* = 10), *P* < 0.05) (Fig. 4D). This presented as significantly larger droplets (0.090 ± 0.008 *μ*m^3^ (*n* = 10) vs. 0.064 ± 0.007 *μ*m^3^ (*n* = 10), *P* < 0.05) in higher number (0.062 ± 0.008 (*n* = 10) vs. 0.031 ±0.006 n/*μ*m^3^ (*n* = 10), *P* < 0.01) (Fig. 4E–F).

#### Electrophysiological and mechanical function in right ventricular tissue strips

**Figure 5:**
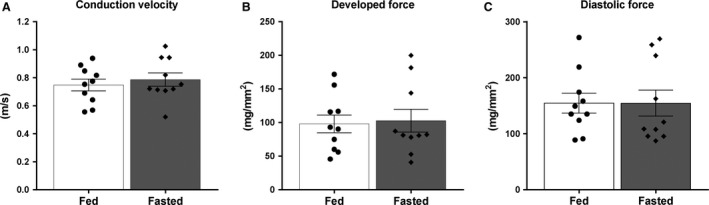
Ventricular longitudinal conduction velocity, developed force, and diastolic force in fed and fasted rats. (A) Conduction velocity, (B) developed force, and (C) diastolic force was measured in the longitudinal direction of right ventricular strips from rats either fed normal chow ad libitum (*n* = 10) or fasted overnight (*n* = 10). Values are presented as means ± SEM.

Despite the significant increase in triglyceride content and increase in lipid droplet deposition, no difference in longitudinal conduction velocity was observed between fed and fasted rats (0.75 ± 0.042 m/sec (*n* = 10) vs. 0.79 ± 0.047 m/sec (*n* = 10), NS) (Fig. 5A). There was also no difference between fed and fasted rats in developed force (98.0 ± 13.3 mg/mm^2^ (*n* = 10) vs. 102.8 ±17.0 mg/mm^2^ (*n* = 10), NS) (Fig. 5B) or diastolic force (154.7 ± 17.8 mg/mm^2^ (*n* = 10) vs. 154.6 ± 23.1 mg/mm^2^ (*n* = 10), NS) (Fig. 5C).

## Discussion

In this paper, we investigated whether an acute elevation of intramyocardial triglycerides by palmitate‐glucose perfusion or a subacute elevation of triglycerides and lipid droplets by fasting, reduce longitudinal conduction velocity per se. However, no difference in longitudinal conduction velocity was observed for either of the models. We conclude that cardiac steatosis per se does not slow longitudinal conduction velocity within such a short period of time.

### Lipid loading by palmitate‐glucose perfusion

Perfusion with palmitate‐glucose increased the myocardial triglyceride content as expected from previous studies. Saddik and Lopaschuk showed that a palmitate buffer increased the overall triglyceride pool during working heart perfusions (Saddik and Lopaschuk 1994). In this study, we found that mechanical function declined in both palmitate‐glucose and glucose‐perfused hearts and that hearts developed edema during perfusion. Edema is a known complication to Langendorff perfusion that can impair cardiac function (Mehlhorn et al. 2001), and is a likely explanation for the declining mechanical function. In this study, perfusion with palmitate‐glucose did not exert major effect on mechanical function and if anything, slightly increased developed pressure. In contrast, Lopaschuk and coworkers demonstrated that mechanical function was reduced by palmitate perfusion in working heart mode (Saddik and Lopaschuk 1991, 1994). This difference may be explained by the larger workload in the working heart preparation, where metabolizing lipids could increase the oxygen demand beyond the delivery capacity of the perfusion buffer. In any case, this study showed no difference in diastolic‐ or developed force between tissues trips from palmitate‐glucose or glucose‐perfused hearts, and therefore any small differences in mechanical function observed during the Langendorff perfusion had no permanent effects on right ventricular strips.

After the loading protocol, the triglyceride levels in palmitate‐glucose perfused hearts were significantly higher compared to glucose perfused hearts and at a level comparable to that of the fructose‐fat fed rats (Axelsen et al. 2015). Saddik and Lopaschuk also found an expansion of the triglyceride pool when perfusing hearts with 1.2 mmol/L palmitate in one study (Saddik and Lopaschuk 1994), whereas another study showed that palmitate perfusion merely sustained intramyocardial triglyceride pools (Saddik and Lopaschuk 1991). The effect likely depends on the balance between workload and metabolism, and experiments using 0.5 mmol/L palmitate perfusion, demonstrated reduced intramyocardial triglyceride levels after 1 h of perfusion (Paulson and Crass 1982). Perfused hearts likely metabolize internal triglyceride stores when lipid delivery is insufficient, and perfusion with glucose alone reduces the myocardial triglyceride content as demonstrated by Saddik and Lopaschuk (Saddik and Lopaschuk 1991), as well as by this study.

Although the cardiac triglyceride content of palmitate‐glucose‐perfused hearts was higher than that of glucose‐perfused hearts, it did not result in lipid droplet accumulation. The reason for this could be either high turnover of the triglycerides, or that it takes longer time before an increased amount of triglycerides transforms into lipid droplets. The time requirement for lipid droplet formation in the heart is not well described. Lipid droplet formation is a complex process where various proteins assemble small lipid droplets, which subsequently undergo a series of highly regulated fusions before reaching a mature size (reviewed in (Murphy and Vance 1999)). When treating skeletal muscle fibers with a mixture of lipids overnight Spangenburg et al. (2011) observed increased lipid droplet levels and Brasaemle and coworkers also found an increased amount of lipid droplets in fibroblastic adipocytes after 16 h of incubation with oleic acid (Brasaemle et al. 2000). Under stressed conditions, however, this process can accelerate, and Bozza et al. (1996) showed that leukocytes, as well as macrophages, stimulated with unsaturated fatty acids formed detectable new lipid bodies within 1 h. Finally, Hexeberg and colleges reported elevated lipid droplet levels in rabbit hearts after 45 min of in vivo lipid infusion (Hexeberg et al. 1995). In the Langendorff palmitate‐glucose perfused hearts, it is possible that small lipid bodies did in fact form within the 120 min of perfusion, but without having time to fuse to a size that was detectable with our method.

With an increased intramyocardial triglyceride content, it is reasonable to assume that the cardiac cells has taken up a significant amount of palmitate, and therefore also have increased levels of this saturated‐free fatty acid. Fatty acids are shown to decrease membrane excitability (Kang et al. 1995) and shorten action potential duration (Haim et al. 2010) in isolated cardiomyocytes. These findings suggest a direct effect of free fatty acids on the electrophysiology of isolated cells. This study show that palmitate‐glucose perfusion did not alter longitudinal conduction velocity per se, suggesting that free fatty acids are less likely to affect membrane excitability in intact tissue. However, whether palmitate‐glucose perfusion changes action potential morphology in intact cardiac tissue remains uncertain.

Although the triglyceride content in the palmitate‐glucose perfused hearts resembles the content in fructose‐fat fed rats the cardiac lipid droplet profile is very different. We hypothesized that the presence of lipids can affect conduction velocity per se, either by increasing the intracellular resistance, acting as isolators, or via lipotoxic metabolites. Lacking lipid droplets and only presenting increased triglyceride levels, the palmitate‐glucose perfusion model does not fulfill the requirements needed to address the first question. This study show that the mere presence of short‐term triglyceride accumulation is not sufficient to slow longitudinal conduction velocity, and in order to evaluate whether lipid accumulation in form of lipid droplets would slow longitudinal conduction velocity per se, we induced a subacute intramyocardial lipid steatosis by fasting rats overnight.

### Lipid loading by overnight fasting

In accordance with other studies investigating the effect of longer lasting calorie restriction in humans (Reingold et al. 2005; van der Meer et al. 2007; Hammer et al. 2008a,b), rats (Schneider and Taegtmeyer 1991; Schneider et al. 1991) and mice (Suzuki et al. 2002; Han et al. 2004; Kienesberger et al. 2012; Trent et al. 2014), fasting rats overnight increased intramyocardial triglyceride levels compared to controls. Lipid droplet volume also increased in these rats, which has also been observed in mice (Suzuki et al. 2002, 2009; Trent et al. 2014; Ueno et al. 2017).

By evaluating the hearts after overnight fasting, we isolate the component of cardiac lipid steatosis from the other metabolic alterations associated with prediabetes, such as increased fasting blood glucose, hyper‐insulinemia and impaired glucose tolerance, all of which was present in the previously evaluated fructose‐fat fed rats (Axelsen et al. 2010a, 2015). In accordance with others investigating cardiac function after an overnight fast, diastolic force, and developed force was comparable in tissue strips from fed and fasted rats (Schneider and Taegtmeyer 1991; Schneider et al. 1991; Bakermans et al. 2011; Trent et al. 2014). Overnight fasting, as a model with a lipid profile resembling fructose‐fat fed rats, though without long‐term comorbidities, was suitable for investigating the isolated effect of cardiac steatosis on conduction velocity. There was, however, no difference in longitudinal conduction velocity between fasted and fed rats, which all together led to the conclusion that lipid droplet accumulation does not cause conduction slowing per se. Our study therefore suggests that conduction slowing may arise by mechanisms that, despite the correlation with lipid accumulation, is independent thereof; or that lipid accumulation acts by long‐term effects.

### Limitations

Although this study shows that acute lipid loading does not slow longitudinal conduction, we cannot rule out that transverse conduction is affected. Several studies demonstrate that transverse conduction is more sensitive to various pertubations (e.g., (Veeraraghavan et al. 2012; George et al. 2015, 2019)), but a simultaneous measurement of both longitudinal and transverse conduction would require the use of either an electrode grid or optical mapping. These techniques also give a more detailed information about the spatial distribution of conduction velocities, as opposed to obtaining a single value using only two electrodes. Irrespective of this limitation, acute lipid loading did not slow longitudinal conduction velocity, in contrast to our observations in ZDF and fructose‐fat fed rats that exhibit long‐term cardiac steatosis and longitudinal conduction slowing.

## Conclusion

This study shows that lipids in the form of either intramyocardial triglyceride accumulation alone or intramyocardial triglyceride and lipid droplet accumulation together, does not slow longitudinal conduction velocity per se. This does not exclude cardiac steatosis from being involved in the pathology of electrical disturbances on a longer time scale. Since the lipid droplets themselves do not slow conduction, we are left without an explanation as to why cardiac conduction is slowed in the fructose‐fat fed and Zucker diabetic fatty rats. The presented results also leaves us without the complete knowledge of the time‐dependent correlation between development of lipid steatosis and conduction velocity slowing. Hence, determining the timing of conduction velocity slowing and the development of cardiac steatosis could further help clarify whether lipid accumulation precedes the electrical disturbances, and whether more chronic lipotoxic effects are likely to be involved in the mechanism of diabetes associated conduction disturbances in the heart.

## Conflict of Interest

The authors declare that they have no competing interests.
